# Passion for an activity and its role on affect: Does personality and the type of activity matter?

**DOI:** 10.3389/fpsyg.2022.1047257

**Published:** 2023-01-04

**Authors:** Nikolaos Mylonopoulos, Vasilis Theoharakis

**Affiliations:** ^1^Alba Graduate Business School, The American College of Greece, Athens, Greece; ^2^School of Management, Cranfield University, Cranfield, United Kingdom

**Keywords:** social media, physical exercise, personality, passion, affect

## Abstract

While personality traits play a crucial role in a person’s general affect, passion for an activity has been shown to partially mediate this relationship, with harmonious passion generally related to positive affect and obsessive passion to negative affect. However, activities are not all the same with some characterized as having “positive” consequences while others as having “negative” consequences. This study examines how passions manifest for two popular activities: physical exercise, an activity with in general “positive” consequences, and social media, an activity with potentially both “positive” and “negative” consequences. We replicate and extend earlier studies which have relied on baskets of heterogenous self-reported activities without distinguishing between activities. We find that, when fully controlling for personality, obsessive passion for physical exercise is positively associated with positive affect while obsessive passion for social media is positively associated with negative affect. However, harmonious passion for either activity has no significant association with any affect. Further, we find that passions for physical exercise relate with conscientiousness while passions for social media with neuroticism.

## Introduction

The dualistic theory of passions describes the strong inclination toward an activity which people find important, engage with, investing time and energy, and which ultimately becomes part of the person’s identity and is manifested as harmonious and obsessive passion (Vallerand et al., 2003). In particular, harmonious passion is defined as a mode of engagement with an activity in congruence with life objectives and is expected to minimize the experience of negative affect. In contrast, obsessive passion is defined as an overpowering compulsion that poisons one’s life, therefore leading to negative affect. These relationships between passions and affect are supported by a recent study by Dalpé et al. (2019) which partially controlled for personality traits while relying on a basket of self-reported heterogeneous activities.

In this paper we seek to replicate and extend Dalpé et al. (2019) as they have not fully controlled for the influence of all personality traits on affect (e.g., neuroticism on negative affect or extraversion on positive affect). This omission raises concerns since the Big Five personality traits (Neuroticism, Extraversion, Agreeableness, Openness to Experience, and Conscientiousness) have been repeatedly found to be stronger predictors of affect over other variables such as demographics or culture (Diener, 2000; Lucas and Diener, 2009; Diener et al., 2018), accounting for 39–63% of the total variance for subjective well-being (Steel et al., 2008). More specifically, neuroticism is reported to have a positive influence on negative affect, a relationship that is well stablished in the literature (Costa and McCrae, 1980), and extraversion and conscientiousness are reported to strongly correlate with positive affect (Anglim et al., 2020). Therefore, the first objective of this replication is based on our concern about fully controlling for all personality traits.

The second objective of this replication is to examine how passion for different activities influences affect in distinct ways. According to Vallerand (2012), activities can be generally seen as “positive” (e.g., yoga) or “negative” (e.g., gambling) depending on their consequences on well-being, which suggests that the relationship between passion and affect may vary depending on the activity. Studies on the relationship between personality, passions, and affect (Balon et al., 2013; Schellenberg and Bailis, 2015; Dalpé et al., 2019) ask participants to select their favorite activity and then tend to treat the heterogeneous activities reported as one basket, without considering the possibility that passions for different activities may manifest themselves differently. Furthermore, studies based on baskets of self-reported favorite activities may hide systematic bias, such as self-enhancing identity motivations (Gregg et al., 2011) according to which participants are likely to under-report activities such as gambling (Mageau et al., 2005) that may be less congruent with their positive self-image. This would imply that a greater proportion of “positive” self-enhancing activities are reported, which may relate differently to affect, versus “negative” activities that may appear as self-harming to the person’s identity.

Therefore, this paper takes a different approach than Dalpé et al. (2019), who studied self-reported “favorite” activities, and excluded nearly a quarter of responses where the reported activities were deemed “inappropriate.” While Dalpé et al. (2019) find harmonious passion to be associated with positive affect and obsessive passion with negative affect, in this study we suggest that obsessive passion may also have positive consequences (e.g., Vergauwe et al., 2022). This argument is supported by an extensive meta-analysis which paints a more equivocal picture where the activity domain emerges as a significant moderator for the consequences of passions (Curran et al., 2015). Similarly, Vallerand et al. (2007) find mixed evidence with regards to obsessive passion and subjective well-being, while Schellenberg and Bailis (2015) find that obsessive passion for two self-reported favorite activities may be related to higher positive affect as compared to having no passions.

The third objective for this replication is based on the observation that personality traits orient people toward the choice of activities they select (Furnham, 1981; Stephan et al., 2014; Wilt and Revelle, 2019) and they passionately dedicate themselves to (Vallerand, 2012). Therefore, the aggregation of heterogeneous activities into a single basket overlooks the potentially different relationship between personality traits and the selection of passionate activities. For this reason, in this study we examine how personality may relate differently to passions for distinct activities.

Our empirical research design focuses on two popular activities, namely physical exercise and social media use since physical exercise is the most frequently self-reported activity in previous studies (Balon et al., 2013; Schellenberg and Bailis, 2015; Dalpé et al., 2019) and roughly half the world’s population (Statista, 2021) spends more than 2 hours daily on social media (Armstrong, 2021) even if they are not frequently self-reported as a favorite activity in passion research.

Physical exercise is broadly viewed as an activity with “positive” consequences as it improves well-being (Netz et al., 2005), despite the relatively rare cases where extreme commitment to physical exercise can have serious negative effects (Szabo, 2000). In the context of amateur and professional sports, Vallerand and Verner-Filion (2020) observe that obsessive passion may be more conducive to higher performance than harmonious passion, as it imposes persistence against adverse or risky conditions (e.g., bad weather, risk of injury). For this reason we expect obsessive passion for physical exercise to be associated with positive affect.

In contrast, while obsessive passion for physical exercise may be seen as “positive,” this may not be the case with social media which have been related to problematic use (Cataldo et al., 2021). More specifically, obsessive passion for social media leads to higher levels of use (Mylonopoulos and Theoharakis, 2020) and correlates with negative consequences such as addiction-like symptoms (Mylonopoulos and Theoharakis, 2021). While experts caution against over-pathologizing social media use (Billieux et al., 2015) and raise concerns about measurement shortcomings related to the psychological impact of social media (Davidson and Ellis, 2019), positive outcomes of social media use such as enhanced socializing and recreation have also been identified (Wang et al., 2015). However, positive outcomes are associated with the routine use of social media, while negative outcomes are associated with a stronger emotional investment to social media (Bekalu et al., 2019). This finding echoes the distinction between harmonious passion as routine self-controlled social media use associated with positive affect versus obsessive passion with social media entailing a stronger emotional investment which is potentially associated with negative affect. In summary, we expect that the relationship between passions and affect in the case of social media will not be the same as in the case of physical exercise.

Furthermore, given that passions represent the internalization of an activity into a person’s identity commensurate with their degree of commitment and engagement with the activity (Vallerand et al., 2003), we expect the relationship between personality traits and harmonious and obsessive passions to vary depending on the activity. In particular, physical activity tends to positively relate to openness, extraversion and conscientiousness, but negatively relate to neuroticism, and does not relate to agreeableness (Wilson and Dishman, 2015). However, the use of social networks tends to positively relate to neuroticism (Yu et al., 2020; Cataldo et al., 2021; Bowden-Green et al., 2021b) and extraversion (Bowden-Green et al., 2020) and does not relate to the other three personality traits (Liu and Campbell, 2017).

Overall, we examine how the relationships between personality, passion and affect may depend on activity type by first replicating and then extending the Dalpé et al. (2019) study to pursue the following questions: (i) how do personality traits relate with passion for the two activities? (ii) how does controlling for personality influence the relationship between passions and affect? (iii) how does the relationship between personality, passion, and affect differ when considering individual activities separately without relying on self-reported baskets of heterogenous activities?

## Materials and methods

### Participants and procedures

For the data collection, we utilized Pollfish, an online survey platform which delivers surveys globally, primarily on users’ mobile phones^1^ *via* a panel of over 250 million people and has been previously demonstrated to be quite representative of the population (Goel et al., 2015)^2^. While the Pollfish platform is governed by a strict privacy policy,^3^ this study also received approval by the research ethics committee of the author’s institution. No compensation was offered by the researchers and users could opt out at any point of the survey. The survey was completed by 310 individuals based in the United Kingdom (133 males and 177 female), aged from 18 to 77 years old (Mean = 40.01 SD = 13.04).

### Measures

#### Personality traits

Personality traits were measured based on the International Personality Item Pool [IPIP; (Goldberg et al., 2006)]. Given that the Pollfish platform limits users to a total of 50 survey questions, we had to restrict our measurement of personality traits to 15 items (i.e., 3 per trait). The specific items used were primarily drawn from the positive keyed items listed as similar to the NEO-PI-R on the IPIP Pool website.^4^ More specifically, we used items “Skilled in handling social situations,” “Feel comfortable around people,” and “Make friends easily” for extraversion, “Have a vivid imagination,” “Open to new experiences,” and “Enjoy hearing new ideas” for openness to experience, “Have a good word for everyone,” “Respect others,” and “Accept people as they are” for agreeableness, “Get chores done right away,” “Carry out my plans,” and “Make plans and stick to them” for conscientiousness, and “Feel often down in the dumps,” “Have frequent mood swings,” and “Panic easily” for neuroticism. Participants responded on a seven-point Likert scale ranging from 1 (“strongly disagree”) to 7 (“strongly agree”) for the five personality dimensions (extraversion α = 0.84, openness to experience α = 0.72, agreeableness α = 0.72, conscientiousness α = 0.71, neuroticism α = 0.83).

#### Passions

All passion measures were assessed based on the scale developed by Vallerand et al. (2003). A seven-point Likert scale ranging from 1 (“strongly disagree”) to 7 (“strongly agree”) was utilized in order to assess harmonious passion (e.g., “[Using social media/Doing physical exercise] allows me to live a variety of experiences”; social media α = 0.86, physical exercise α = 0.92) and obsessive passion (e.g., “The urge is so strong. I cannot help myself from [using social media/doing physical exercise]”; social media α = 0.92, physical exercise α = 0.91) for the two activities selected.

#### Positive and negative affect

We focus on positive and negative affect which are distinct factors and not polar opposites (Diener, 2000). The Positive and Negative Affect Schedule (PANAS) Short Form scale was used (Thompson, 2007) to measure positive (e.g., active, alert, inspired, determined, attentive; α = 0.79) and negative affect (e.g., afraid, nervous, upset, hostile and ashamed; α = 0.82) on a five-point Likert scale ranging from 1 (“never”) to 5 (“always”).

## Results

We separately estimate our models for each activity (Models 1a and 1b, Table 1) by first replicating the exact model of Dalpé et al. (2019). Then we proceed by accounting for all personality traits on passions and affect and adding a path between obsessive passion and positive affect (Models 2a and 2b, Table 1). The descriptive statistics and correlations for the study variables are presented in Table 2. One can observe that positive affect positively relates with all traits except neuroticism where the relationship is negative. At the same time, harmonious passions (HP) for both activities and the obsessive passion (OP) for physical exercise have a positive association with positive affect, indicating that the manifestation and role of obsessive passion does vary depending on the activity. Nonetheless, similarly with Dalpé et al. (2019), negative affect is positively related with neuroticism and with the two obsessive passions, but there are differences in the relationship with other traits. Given the strong bivariate correlations between personality, passions and affects, we examine the relationships with every personality trait in our Structural Equation Models.

**Table 1:** Standardized path coefficients for SEM models of the relationship between personality, passions, and affect for social media and exercise.

	Dalpé et al. (2019) results	SOC model 1a	PE model 1b	SOC model 2a	PE model 2b
Consc → HP	0.27**	0.08	0.09	0.09	0.21**
Open → HP	0.21**	0.27**	0.26**	0.26*	0.31**
Agree → HP	0.15*	−0.01	−0.02	−0.01	−0.09
Extra → HP	0.11	0.23**	0.28**	0.26**	0.24**
Neuro → HP				0.15*	0.09
Consc → OP				−0.00	0.19**
Open → OP				−0.03	0.10
Agree → OP	−0.12	−0.02	−0.09	−0.02	−0.19*
Extra → OP	0.19*	0.33**	0.45**	0.35**	0.38**
Neuro → OP	0.32**	0.38**	0.23**	0.42**	0.25**
HP → PosAff	0.45**	0.16*	0.42**	0.10	0.18
OP → PosAff				−0.03	0.24*
HP → NegAff	−0.21**	−0.24**	−0.29*	−0.10	−0.17
OP → NegAff	0.21**	0.52**	0.40**	0.21*	0.16
Consc → PosAff	0.22**	0.33**	0.26**	0.27**	0.20**
Open → PosAff				0.25**	0.20**
Agree → PosAff				0.08	0.14
Extra → PosAff	0.18**	0.32**	0.24**	0.23**	0.12
Neuro → PosAff	0.14	−0.16*	−0.15*	−0.10	−0.17**
Consc → NegAff	−0.22**	−0.06	−0.09	−0.06	−0.06
Open → NegAff				0.18**	0.18**
Agree → NegAff	−0.18**	0.01	0.06	0.01	0.03
Extra → NegAff				−0.11	−0.08
Neuro → NegAff				0.63**	0.68**

SOC, social media; PE, physical exercise; HP, harmonious passion; OP, obsessive passion; Consc, conscientiousness; Open, openness; Agree, agreeableness; Extra, extraversion; Neuro, neuroticism; PosAff, positive affect; NegAff, negative affect. Model 1 replicates the Dalpé et al. (2019) structural model; Model 2 adds the remaining direct paths from personality traits to passions and affect, and from OP to PosAff. ^*^p < 0.01, two-tailed. ^**^p < 0.001, two-tailed.

**Table 2:** Descriptive statistics and correlations (*N* = 310).

	*M*	*SD*	1	2	3	4	5	6	7	8	9	10	11	12
1. Neuroticism	4.29	1.53												
2. Extraversion	4.85	1.30	−0.18^⁎^											
3. Agreeableness	5.65	0.86	−0.03	0.43^⁎⁎^										
4. Openness	5.40	0.94	−0.03	0.42^⁎⁎^	0.44^⁎⁎^									
5. Conscientiousness	5.14	1.05	−0.07	0.37^⁎⁎^	0.41^⁎⁎^	0.35^⁎⁎^								
6. HP (social media)	4.85	1.26	0.08	0.34^⁎⁎^	0.22^⁎⁎^	0.35^⁎⁎^	0.24^⁎⁎^							
7. OP (social media)	3.58	1.74	0.34^⁎⁎^	0.24^⁎⁎^	0.10	0.08	0.08	0.55^⁎⁎^						
8. HP (phys. exercise)	4.88	1.46	−0.02	0.38^⁎⁎^	0.23^⁎⁎^	0.43^⁎⁎^	0.35^⁎⁎^	0.37^⁎⁎^	0.13					
9. OP (phys. exercise)	3.57	1.64	0.16^⁎^	0.34^⁎⁎^	0.08	0.22^⁎⁎^	0.26^⁎⁎^	0.25^⁎⁎^	0.31^⁎⁎^	0.68^⁎⁎^				
10. Negative affect	3.34	0.67	0.65^⁎⁎^	−0.14	−0.01	0.08	−0.07	0.09	0.31^⁎⁎^	0.02	0.15^⁎^			
11. Positive affect	2.52	0.75	−0.15^⁎^	0.47^⁎⁎^	0.39^⁎⁎^	0.46^⁎⁎^	0.46^⁎⁎^	0.28^⁎⁎^	0.08	0.50^⁎⁎^	0.42^⁎⁎^	−0.04		
12. Age	40.01	13.04	−0.21^⁎⁎^	0.10	−0.06	−0.04	−0.05	0.01	−0.12	−0.09	−0.09	−0.23^⁎⁎^	0.03	
13. Gender^a^	1.57	0.50	0.29^⁎⁎^	−0.09	0.09	−0.02	0.03	−0.07	0.04	−0.10	−0.03	0.16^⁎^	−0.01	−0.02

HP, harmonious passion; OP, obsessive passion. ^a^1, men; 2, women. ^⁎^*p* < 0.01, two-tailed. ^⁎⁎^*p* < 0.001, two-tailed.

### Model evaluation

We utilized the structural equation modeling command available in Stata 17.1. Prior to our data analysis we tested for the assumption of multivariate normality using the mvtest procedure in Stata. Mardia’s test suggested that the data did not completely satisfy the multivariate normal assumption and therefore we used robust maximum likelihood estimation methods with Satorra-Bentler corrections (Savalei, 2018). We initially replicated the model of Dalpé et al. (2019) for each one of our activities (Models 1a and 1b) and then added all other paths, per our conceptual model (Models 2a and 2b) with results available in Table 1. The first set of models yielded fit results that according to Marcoulides and Yuan (2016) would be considered as poor to fair (Model 1a: Satorra-Bentler χ^2^(208) = 439.22, χ2/df = 2.11, CFI = 0.92, RMSEA = 0.060, SRMR = 0.076 and Model 1b: Satorra-Bentler χ^2^(208) = 567.16, χ^2^/df = 2.72, CFI = 0.89, RMSEA = 0.075, SRMR = 0.093) while our final set of models produced fits that are considered to be close (Model 2a: Satorra-Bentler χ^2^(199) = 288.91, χ^2^/df = 1.45, CFI = 0.97, RMSEA = 0.038, SRMR = 0.039 and Model 2b: Satorra-Bentler χ^2^(199) = 381.73, χ^2^/df = 1.91, CFI = 0.94, RMSEA = 0.055, SRMR = 0.056). This indicates that the additional direct effects of personality traits on affect and passions as well as the additional path between obsessive passion and positive affect are important for improving model fit.

Our results show that our first models perfectly replicate the Dalpé et al. (2019) model in the relationship between passion and affects (Model 1a and 1b, Table 1). More specifically, harmonious passion for both activities is associated with positive affect and restrains negative affect, while obsessive passions are associated with negative affect. However, when we fully control for all personality traits and also add the direct effect between OP and positive affect (Models 2a and 2b, Table 1), model results change significantly. In the case of physical exercise, the additional path between OP and positive affect is significant and positive while other paths become non-significant. In the case of social media, we observe that obsessive passion is the only significant influence on negative affect while harmonious passion does not appear to play a significant role (Model 2a). Additional tests indicate that the positive relationship between social media HP and positive affect (Model 1a) is suppressed in the presence of openness to experience (Model 2a). Similarly, the negative relationship between social media HP and negative affect (Model 1a) is suppressed in the presence of either extraversion or neuroticism (Model 2a). This clearly demonstrates that personality plays a key role when examining the relationship between passions and affect requiring that one considers all the relevant traits.

Moreover, we find differences in the relationship between personality and the passions for our two activities. Most notably, social media HP is associated with neuroticism (not the case for physical exercise) while social media OP is more significantly related (*p* < 0.017) to neuroticism (β = 0.42, *p* < 0.001) than physical exercise OP (β =0.25, p < 0.001). As one might expect, conscientiousness is related with both physical exercise passions, but is unrelated with both social media passions.

## Discussion

This paper improves our understanding of the relationship between personality, passions, and affect by examining two activities, one with, in general, “positive” consequences and another with potentially both “negative” and “positive” consequences. It achieves this by replicating and extending recent research (Dalpé et al., 2019) and contributes to the relevant literature in three main ways.

First, personality traits relate differently to the passions for our two activities. On one hand, the contribution of conscientiousness in physical exercise is widely reported (Wilson and Dishman, 2015), and is associated with both physical exercise passions (de Bruijn et al., 2009), while it is unrelated to social media passions. These results might be explained by the fact that physical exercise depends on characteristics related to conscientiousness such as deliberate planning, achievement-striving, and the self-discipline to dutifully carry out one’s plans (Rhodes and Boudreau, 2017), whereas engagement with social media is often unplanned and requires little deliberate effort as users seek to pass time, entertain themselves, relax (Whiting and Williams, 2013) or escape (Orchard et al., 2014). On the other hand, neuroticism relates to both social media passions, which is consistent with recent research arguing that neuroticism is related with greater immersion in social media (Yu et al., 2020) and that concerns about negative social media consequences such as addiction-like symptoms “are particularly pertinent for those with high trait neuroticism” (Bowden-Green et al., 2021a, p. 10). Although neuroticism is also related to the obsessive passion for physical exercise, this association is significantly smaller than that for social media. Moreover, a recent study described the case of healthy neuroticism as long as neuroticism is accompanied by high conscientiousness (Stieger et al., 2020), which is consistent with our results for physical exercise. Extraversion is associated with both passions for both activities (Bowden-Green et al., 2020), while the relation between openness and harmonious passion for both activities is consistent with earlier research (Dalpé et al., 2019). Overall, these results indicate that while both activities do share associations with some personality traits, their main difference is that physical exercise relates with conscientiousness whereas social media engagement relates more strongly to neuroticism. While being consistent with extant research (Wilson and Dishman, 2015; Stieger et al., 2020) our results demonstrate the nuanced relationship between personality and passions depending on the activity.

Second, given the strong relationship between personality and subjective well-being (Diener, 2000), we examine the relationship between passions and affect by controlling for all personality traits. Initially, we successfully replicated previous studies by modelling an incomplete selection of personality traits with no direct paths between obsessive passion and positive affect (Dalpé et al., 2019). However, in our full model, i.e., after controlling for the effect of all personality traits on both types of affect, obsessive passion for physical exercise only has a positive association with positive affect whereas obsessive passion for social media only has a positive association with negative affect. These results highlight the different nature of the two activities and the role of personality in shaping passions. More specifically, the non-significant relationship of physical exercise obsessive passion with negative affect is unexpected because obsessive passion has a significant correlation with neuroticism, which has predicted negative affect in previous studies (Costa and McCrae, 1980). However, this result might be due to the ameliorating role of conscientiousness on the individual’s obsessive passion for exercise, perhaps a case of “healthy neuroticism” (Stieger et al., 2020), which is not present in the case of social media. For example, as pointed out by Vallerand and Verner-Filion (2020), obsessive passion imposes persistence to the pursuit of physical exercise which further augments the secretion of endorphins in the brain causing general euphoria (Dinas et al., 2011), a phenomenon known as the “endorphins hypothesis.” Our result is also consistent with the positive correlation between obsessive passion for yoga and positive emotions (and no correlation with negative emotions) in a longitudinal study by Carbonneau et al. (2010).

In contrast, in the presence of all personality traits, only obsessive passion for social media promotes negative affect, suggesting that social media appears to have some of the makings of a “negative” activity (Vallerand, 2012). Further, while we confirmed that harmonious passion for both activities is positively related to positive affect and negatively related to negative affect when replicating the exact Dalpé et al. (2019) model, once we fully control for all personality traits, these effects are no longer statistically significant. This indicates that individual differences have a stronger explanatory power than harmonious passion.

Third, we demonstrate that relying on self-reported baskets of heterogeneous activities (Balon et al., 2013; Schellenberg and Bailis, 2015; Dalpé et al., 2019) is problematic as the passion for different activities relates differently with personality and affect. While such baskets may over-represent self-enhancing activities such as physical exercise, they under-represent the reporting of other activities such as drinking, smoking, gambling, or social media that may be perceived as harmful to one’s sense of identity. In any case, the aggregation of different activities results in significant loss of information that is specific to the activity. For example, in the context of multiplayer online games, Mandryk et al. (2020) found wellbeing to be influenced by harmonious passion and not by obsessive passion and it remains to be seen if the results would be different when controlling for personality traits. In another example, similarly with our results for physical exercise, Vergauwe et al. (2022) found passion for work to relate with conscientiousness and obsessive passion for work to positively relate with job satisfaction. Therefore, this study demonstrates that the dualistic theory of passions offers a far more nuanced insight and clarity when the distinctive characteristics of activities are examined.

To conclude, this study unpacks the idiosyncratic role of passions for two different, yet popular, activities and remains consistent with Vallerand (2015) who points out that passion is a function of the interaction between the activity (whether it allows the person to satisfy basic psychological needs), the environment (the extent to which it encourages autonomy), and the person (their personality). Our results indicate that only obsessive passion partially mediates personality traits and lend support to the twin argument that (1) the nature of the activity can make a significant difference in the manifestation and consequences of passions, and (2) to discern the role of passions, studies must comprehensively account for the influence of personality traits, as the latter are closely related to activity choice and general affect.

### Limitations and future research

Even though this paper replicated and significantly extended prior studies, its research design has imposed certain limitations and presents opportunities for further research. First, a longitudinal or experimental research design could enhance our understanding of the causal relationship between passions and affect, since it has been argued that a general state of depression might push people to spend more time on social media rather than the other way around (Hartanto et al., 2021). Second, further extensions to this line of research could examine a wider range of activities, possibly leading to the identification of more refined activity type categories (beyond “positive” and “negative”). Even though personality, passions, and well-being have each been separately studied in the context of a great variety of activities (related to work or leisure), there is a paucity of research examining the relationships between all three in the context of individual activities. Third, similar studies should examine other measures of personality and affect, as well as other constituents of subjective well-being, such as general life satisfaction or job satisfaction. Fourth, a question that naturally emerges from these findings is whether the passion for an activity like physical exercise might counteract the apparent negative consequences of an activity like social media. In other words, can developing a passion for an activity with “positive” consequences undo the harm of the passion for an activity with “negative” consequences? Fifth, our findings indicate that the role of harmonious passion needs to be further investigated. On one hand, when we control for personality traits, it is not associated with positive or negative affect as suggested by previous studies. On the other hand, there is very little prior research on the relationship between neuroticism and harmonious passion.

## Data availability statement

The original contributions presented in the study are included in the article/supplementary material, further inquiries can be directed to the corresponding author v.theoharakis@cranfield.ac.uk.

## Ethics statement

The studies involving human participants were reviewed and approved by Cranfield University. The patients/participants provided their written informed consent to participate in this study.

## Author contributions

All authors listed have made a substantial, direct, and intellectual contribution to the work and approved it for publication.

## Conflict of interest

The authors declare that the research was conducted in the absence of any commercial or financial relationships that could be construed as a potential conflict of interest.

## Publisher’s note

All claims expressed in this article are solely those of the authors and do not necessarily represent those of their affiliated organizations, or those of the publisher, the editors and the reviewers. Any product that may be evaluated in this article, or claim that may be made by its manufacturer, is not guaranteed or endorsed by the publisher.

## Footnotes

^1^ www.pollfish.com

^2^ Goel, S., Obeng, A., and Rothschild, D. (2015). *Non-representative surveys: fast, cheap, and mostly accurate in working paper*. Available at: https://researchdmr.com/FastCheapAccurate.pdf

^3^ https://www.pollfish.com/terms/respondent

^4^ https://ipip.ori.org/newNEOKey.htm
